# Hemoperitoneum After Spontaneous Rupture of Liver Tumor: Results of Surgical Treatment

**DOI:** 10.1155/1988/58957

**Published:** 1988

**Authors:** Roland Andersson, Karl-Göran Tranberg, Stig Bengmark

**Affiliations:** Department of Surgery Lund University Lund S-221 85 Sweden

## Abstract

Five cases of massive hemoperitoneum caused by spontaneous rupture of liver tumors, collected during a 27-year period, are reported. Four patients had a primary liver malignancy and one patient a liver cyst with hemangioma. Initial symptoms were obscure and hemoperitoneum was suspected pre-operatively in only one patient. At operation, a mean of 3100 ml of blood was found in the abdomen. Hemostatis was achieved by liver resection in four patients and by suture ligation in one. Two patients died during or shortly after operation. The three patients surviving the operation had primary liver cancer and lived for 6 months to 6.5 years. It is concluded that liver resection, whenever possible, is the treatment of choice and that pre-operative delay and mortality may be diminished by increased awareness of this condition.

## INTRODUCTION

Massive hemoperitoneum caused by spontaneous rupture of the liver is rare. It is usually associated with primary liver cancer, the first case being reported by André in 1851[1]. This study reports our experience of spontaneous rupture of liver tumors accompanied by massive bleeding into the peritoneal cavity.

## MATERIAL

Between 1960 and 1986, a total of five patients were operated because of spontaneous rupture of a liver tumor with intraperitoneal bleeding. Three patients were male and two female, with a mean age of 66 (range 55 – 78) years. Four patients had hepatocellular carcinoma, varying in diameter from 10 to 15 cm, and one patient had a liver cyst combined with hemangioma. Two of the four patients with hepatocellular carcinoma also had liver cirrhosis. Four of the lesions were located in the right liver lobe and one in the caudate lobe. The liver disease was unknown before surgery in all cases.

## RESULTS

Four patients presented with a systolic blood pressure at or below 80 mmHg. A

sudden onset of symptoms was obligatory. Abdominal pain was always present, localized in the upper right quadrant ($n$=2) or diffuse in between the upper abdomen and the thorax ($n$=3). However, symptoms such as dyspnoe, nausea, dizziness or thoracic pain were predominant in three patients, who on admission were considered to suffer from myocardial infarction or cardiac insufficiency. After resuscitation, these patients were referred to the surgical department because of signs of peritonitis and increasing abdominal pain.

The interval between admission to the hospital and start of surgery was 1 – 12 h (median 4 h). Pre-operatively a perforated peptic ulcer was suspected in three patients and intra-abdominal bleeding in one. At operation a mean of 3100 (range 1500–5000) ml of blood was found within the abdominal cavity. Hemostasis was achieved by right hepatectomy ($n$=2), wedge resection ($n$=2) or suture ligation ($n$=1).

The first two patients treated had 4000 – 5000 ml of blood in the abdomen and died intra-operatively or early postoperatively. One of these patients had a liver cyst with hemangioma and was treated with hepatectomy, whereas the other patient had a hepatoma in a cirrhotic liver and was treated with suture ligation for control of bleeding. The remaining three patients, all with hepatocellular carcinoma, had an uneventful postoperative course and were discharged after a mean hospital stay of 16 (range 14–20) days. One patient died of recurrent disease 6.5 years after the operation, one patient was symptom free six months after operation and then lost to follow-up, while the third patient was well six months postoperatively.

## DISCUSSION

Spontaneous rupture of a liver tumor with hemoperitoneum is rare and is most frequently seen in association with liver cell adenoma or primary liver cancer. It is extremely uncommon in liver metastases[2], which tend to be more fibrotic and less vascularized[3,4]. We have not seen any patient with intraperitoneal rupture of a bleeding benign adenoma of the liver during the period studied, and this may be due to the rarity of this disease in Sweden.

Usually the diagnosis is not made until operation or autopsy[5]. The difficulty in obtaining a correct pre-operative diagnosis is demonstrated also in our study, perforated ulcer being the most common pre-operative diagnosis. Operation was delayed in three patients because initial symptoms were vague and more suggestive of cardiac disease than intraperitoneal disease with bleeding. Increased use of emergency ultrasonography or diagnostic laparocentesis in patients with a sudden onset of vague signs of circulatory shock and upper abdominal or lower thoracic pain may lead to earlier diagnosis and improved outcome.

Many ways of obtaining control of the hemorrhage have been described, including liver resection, excision, suture ligation, packing or hepatic artery ligation[4,7–10]. Liver resection is the treatment of choice in a patient with tumor if this is technically feasible and co-existent cirrhosis does not preclude resection[5,11]. In contrast, the management of hemoperitoneum after liver trauma is more conservative, and increasingly so during the last years[12–14], and liver resection is only rarely (about 5% of all cases with liver trauma) necessary or advisable in this situation. If the liver malignancy is previously known and the hemoperitoneum can be verified by diagnostic procedures, an emergency hepatic angiogram with transcatheter arterial embolization may be tried for hemostasis[15].

Provided the surgical procedure is uneventful, the prognosis is, of course, excellent when the intraperitoneal bleeding is due to a liver cell adenoma or another non-malignant tumor. With respect to ruptured hepatocellular carcinoma, Ong *et al.*[6] reported that 18 of 20 patients were dead within six months. Similar results were reported by Mokka *et al.*[7]: their five patients left the hospital alive but only one survived six months. However, our series demonstrates that surgery may result in worthwhile long-term palliation also in patients with hemoperitoneum after spontaneous rupture of primary liver cancer.
